# A systematic review of outcomes reported in randomized controlled trials involving people with patellar dislocations

**DOI:** 10.1302/2633-1462.69.BJO-2025-0045.R1

**Published:** 2025-09-04

**Authors:** Colin P. Forde, Crispin Mortimer, Toby O. Smith, Matthew L. Costa, Jonathan A. Cook, Elizabeth Tutton, Georgina Wistow, Paul Minty, David J. Keene

**Affiliations:** 1 Oxford Trauma and Emergency Care, Nuffield Department of Orthopaedics, Rheumatology and Musculoskeletal Sciences, University of Oxford, Oxford, UK; 2 Physiotherapy Department, Tavistock Hospital, University Hospitals Plymouth, Plymouth, UK; 3 Warwick Clinical Trials Unit, Warwick Medical School, University of Warwick, Coventry, UK; 4 Oxford Clinical Trials Research Unit, Centre for Statistics in Medicine, Nuffield Department of Orthopaedics, Rheumatology and Musculoskeletal Sciences, University of Oxford, Oxford, UK; 5 Physiotherapy Department, John Radcliffe Hospital, Oxford University Hospitals NHS Foundation Trust, Oxford, UK; 6 Therapies Department, Royal United Hospitals Bath NHS Foundation Trust, Bath, UK

**Keywords:** Patellar instability, Randomized controlled trials, Core outcome set, Patellar dislocation, Outcomes, patellar dislocations, randomized controlled trials, patient-reported outcome measures (PROMs), recurrent patellar dislocations, Cochrane Database, CINAHL, MEDLINE, Knee injury and Osteoarthritis Outcome Score, Knee, visual analogue scale

## Abstract

**Aims:**

The primary aims were to determine what outcome domains, outcome measurement instruments, and outcome measurement timepoints are reported in randomized controlled trials (RCTs) involving people with patellar dislocations. The secondary aims were to determine what primary outcomes were used and how a recurrent patellar dislocation was defined when this was used as an outcome.

**Methods:**

We searched MEDLINE, Embase, CINAHL, the Cochrane Database of Controlled Trials, and trial registries (last search: January 2024) for RCTs evaluating treatments for people with a patellar dislocation irrespective of age or sex. We identified the unique outcomes in included studies and mapped these onto the World Health Organization’s International Classification of Functioning, Disability and Health (WHO ICF) framework to identify the measured domains. We synthesized results into tables, figures, and text. A critical appraisal of included studies was not required for this systematic review.

**Results:**

From the 70 included studies, we identified 141 unique outcomes. The most commonly used unique outcome was a recurrent ipsilateral patellar dislocation (used in 55 studies), but only 17/55 studies (31%) reported how this was defined (i.e. the criteria required for a recurrent ipsilateral patellar dislocation event to be recorded). Unique outcomes mapped onto 66 second-level domains of the WHO ICF framework, and 56% (593/1,052) in the ‘activities and participation’ domain. Included studies used 42 different patient-reported outcome measures (PROMs), most commonly the Kujala Patellofemoral Score (71%, 50/70 studies), but 28 PROMs (60%) were used only once. In all, 31 different primary outcomes were identified from 47 included studies, with 14 primary outcomes (45%) used only once among included studies. The Kujala Patellofemoral Score was also the most common primary outcome (38%, 18/47 studies). Outcome measurement timepoints varied, but the most common timeframe for primary outcome measurement was > one to three years (46%, 16/35 studies that provided data).

**Conclusion:**

The variability in the outcome domains, PROMs, and primary outcomes measured in RCTs evaluating patellar dislocation treatments highlights that a core outcome set is needed. This process is underway and is being informed by this systematic review’s findings.

Cite this article: *Bone Jt Open* 2025;6(9):1031–1043.

## Introduction

Patellar dislocations are common knee injuries that primarily affect adolescents and young adults.^1,2^ Treatment is either non-surgical, with the main components usually being advice and exercise-based rehabilitation,^3,4^ or surgical to address the underlying anatomy driving instability symptoms.^5^ However, there is currently insufficient high-quality evidence to guide treatment decision-making for different injury presentations and subgroups of patients, and treatment outcomes can be suboptimal.^4,5^ High-quality randomized controlled trials (RCTs) are needed to address this evidence gap.^6,7^

A key stage in RCT design is outcome selection. Outcomes measure the effects of treatment (i.e. treatment benefits and harms).^8^ Outcome selection in RCTs is crucial because results from these studies inform clinical and policy decision-making. Ideally, RCTs evaluating treatments for the same health condition would measure similar outcomes. This would aid their comparison and the ability to meta-analyse results from individual studies. Meta-analyses can provide more precise treatment effect estimates than individual trials,^9^ potentially providing better evidence to inform healthcare decisions. Despite the benefits of measuring common outcomes, variable outcome selection across studies is common in many clinical areas.^10,11^

Presently, selecting outcomes for RCTs evaluating patellar dislocation treatments is challenging because there is no agreed set of outcomes that should be measured and reported, a so called core outcome set (COS).^12^ Recent systematic reviews have highlighted the need to establish consensus in this area.^4,5^ When developing a COS, a key initial step is to determine what outcomes have already been measured in studies of the health condition.^8^ Therefore, to inform the development of a future COS for trials involving people with patellar dislocations, this systematic review aimed to determine:

1. what was measured (i.e. the outcome domain);
2. how (i.e. the outcome measurement instrument); and
3. when (i.e. the outcome measurement timepoints), in RCTs involving people with patellar dislocations.

Secondary aims were to determine:

1. the primary outcome measures; and
2. how a recurrent patellar dislocation was defined when this was used as an outcome.

## Methods

This systematic review was registered on the Open Science Framework^13^ before title and abstract screening. Changes to the protocol and the rationale for these are presented in Supplementary Table i. Reporting follows the Preferred Reporting Items for Systematic Reviews and Meta-Analyses (PRISMA) guideline^14^ and its extension for reporting literature searches.^15^ No ethical approval or informed consent was required for this systematic review.

### Search strategy

We searched MEDLINE (Ovid), Embase (Ovid), CINAHL (EBSCOhost), the Cochrane Database of Controlled Trials (Cochrane Library), and the following trial registries: ClinicalTrials.gov, the International Standard Randomized Controlled Trial Number (ISRCTN) registry, and the World Health Organization (WHO) International Clinical Trials Registry Platform (ICTRP). We used the Cochrane highly sensitive filter for identifying randomized trials in MEDLINE and Embase, and incorporated the Cochrane filter for identifying randomized trials in CINAHL Plus into the CINAHL search.^16^ No date or language restrictions were applied. The search strategy was adapted from Forde et al^17^ and reviewed by a healthcare librarian. Initial searches were conducted in January 2023 and updated in January 2024 (Supplementary Table ii contains the full search strategy, including exact search dates).

To identify additional potentially eligible studies, one reviewer (CPF) screened all included records and relevant systematic reviews.^4,5,18-21^ Two reviewers (CPF, TOS) familiar with the patellar dislocation literature, also checked their personal files for potentially eligible RCTs.

### Eligibility criteria

Study participants could be any age or sex and receiving any treatment for a first-time or recurrent patellar dislocation or for persistent patellar instability following a previous patellar dislocation. We excluded studies of participants with patellar instability that did not clearly report that participants had a previous patellar dislocation. We also excluded studies of people with a patellar dislocation after knee arthroplasty. Both of these were considered separate conditions.

Only RCTs, including pilot and feasibility RCTs, were eligible. This included studies that claimed to be RCTs but used a pseudorandom participant allocation method (hereafter referred to as quasi-RCTs). There were no restrictions on study setting, status (i.e. ongoing or completed), publication language, or follow-up duration. Journal articles (results and protocols), published abstracts, and trial registry records were eligible.

### Study selection

Retrieved records were exported to Rayyan systematic review software^22^ and Microsoft Excel (only the WHO ICTRP records which could not be exported to Rayann), and deduplicated. Trial registry records of the same study that had different titles or registration identifiers on different trial registries were not considered duplicates because these could contain different data.^23^

After deduplication, two reviewers (from CPF, PM, GW, CM) independently screened each title and abstract for eligibility. The same reviewers then independently screened potentially eligible records’ full texts, or for trial registry records the source trial registration website to access the most current and comprehensive information.^23^ Discrepancies between reviewers were resolved by agreement or consulting a third reviewer (DJK) when required.

### Data extraction

Two reviewers (CPF, CM) independently extracted the following data onto a pilot-tested Microsoft Excel file:

1. Study characteristics: authors, publication year, report type (results journal article, trial registry record, etc), study status, study type (RCT, quasi RCT, pilot/feasibility RCT), country, and interventions.
2. Participant characteristics: sample size, age, sex, and patellar dislocation injury characteristics.
3. Outcomes: outcomes, outcome definitions, outcome measurement instruments, outcome measurement timepoints, primary outcome(s), and primary outcome measurement timepoint(s).

Where necessary, we retrieved outcome information from citations in included studies. To guide data extraction, we used the definitions of an outcome and outcome measurement instrument proposed by Prinsen et al.^24^ For pilot and feasibility RCTs, only clinical outcomes were extracted. For trial registry records, data were extracted from the most recent record on the source trial registry website. If the primary outcome was not specified, the outcome that informed the sample size calculation was designated the primary outcome. We also separated composite outcomes into individual components.

Discrepancies were resolved between data extractors unless there was an obvious error in which case the lead reviewer (CPF) corrected this without discussion. A third reviewer (DJK) was consulted where necessary. The quality of outcome reporting in included studies was poor. This led to unforeseen issues during data extraction. Accordingly, we developed and iteratively refined rules to aid consistent data extraction e.g., where the affected leg was not reported, we assumed patellar dislocations were measured on the ipsilateral not contralateral leg. After resolving data queries, the lead reviewer (CPF) ensured data extraction rules were applied consistently across studies.

### Translation

We translated non-English language records using Google Translate (Google, USA) and extracted data from the translation. This has demonstrated high agreement with data extraction from the original language report by native language speakers.^25^ A native German language speaker verified the translation for the one included German-language record.

### Identifying multiple study reports

The following information (where available) was compared between reports to identify and link multiple reports of the same study: 1) authors’ names; 2) study locations; 3) recruitment time periods; 4) trial registration, ethics, and funding details; 5) participant characteristics; 6) intervention characteristics; and 7) outcomes. If uncertainty remained, we emailed corresponding authors of journal articles or named contacts for trial registry records for clarification. If we received no reply, one follow-up email was sent. Overall, 11 people were emailed and two replied providing clarifying information. Two reviewers (CPF, DJK) decided by consensus whether the remaining reports were a unique study or multiple report. Data from multiple reports of unique studies were extracted separately then combined. If different reports contained additional outcome data, all outcome data were included to account for outcome reporting bias.

### Critical appraisal

This systematic review did not assess treatment effectiveness so a critical appraisal of included studies was not performed.

### Data synthesis

Outcomes can be measured or defined differently across studies, so the first step was to identify outcomes that were unique. Unique outcomes have “original meaning and context”^26^ and were identified following the process described by Young et al.^26^ Initially, we compared extracted verbatim outcomes and their definitions. Identical outcomes and those that differed only by measurement timing or minor spelling differences were considered duplicates and deleted. The remaining outcomes were compared and those that meant the same were grouped under one outcome which was renamed if needed e.g., “thigh hypotrophy” and “quadriceps muscle volume” were grouped and renamed “thigh muscle mass”. Throughout, a guiding principle was that if outcomes would not normally be combined in a meta-analysis they were considered unique. Two reviewers (CPF, CM) independently identified and renamed unique outcomes then resolved discrepancies by discussion. Unique outcomes were then circulated to the other reviewers and refined following their feedback.

To identify the measured domains, the lead reviewer (CPF) mapped unique outcomes onto the second-level classification of the WHO International Classification of Functioning, Disability and Health (ICF) framework^27^ using methods adapted from those reported by Cieza et al.^28^ If a unique outcome was a patient-reported outcome measure (PROM) or assessed by a PROM, individual PROM items were mapped onto the WHO ICF. Uncertainties were resolved by consensus among four reviewers (CPF, TOS, DJK, GW).

The synthesis of outcome measurement instruments was restricted to PROMs because these are most likely to inform outcome measurement instrument selection in future trials. PROMs were grouped into ‘region specific’ (lower limb), ‘condition specific’ (patellar dislocation), ‘generic’, and ‘other’ categories. Study-specific PROMs or those deemed unreproducible were excluded from the synthesis because these are unlikely to be widely used in future.

Outcome measurement timepoints were synthesised into time periods adapted from Miller et al,^10^ using the measurement timepoints per study. For example, if outcome A was measured at three months and outcome B was measured at three and six months, three and six months were only counted once in the synthesis.

Results are presented in tables, figures, and text. Quantitative data were analyzed using appropriate descriptive statistics (means and SDs, medians and IQRs, and percentages).

## Results

### Study selection

**Fig. 1:**
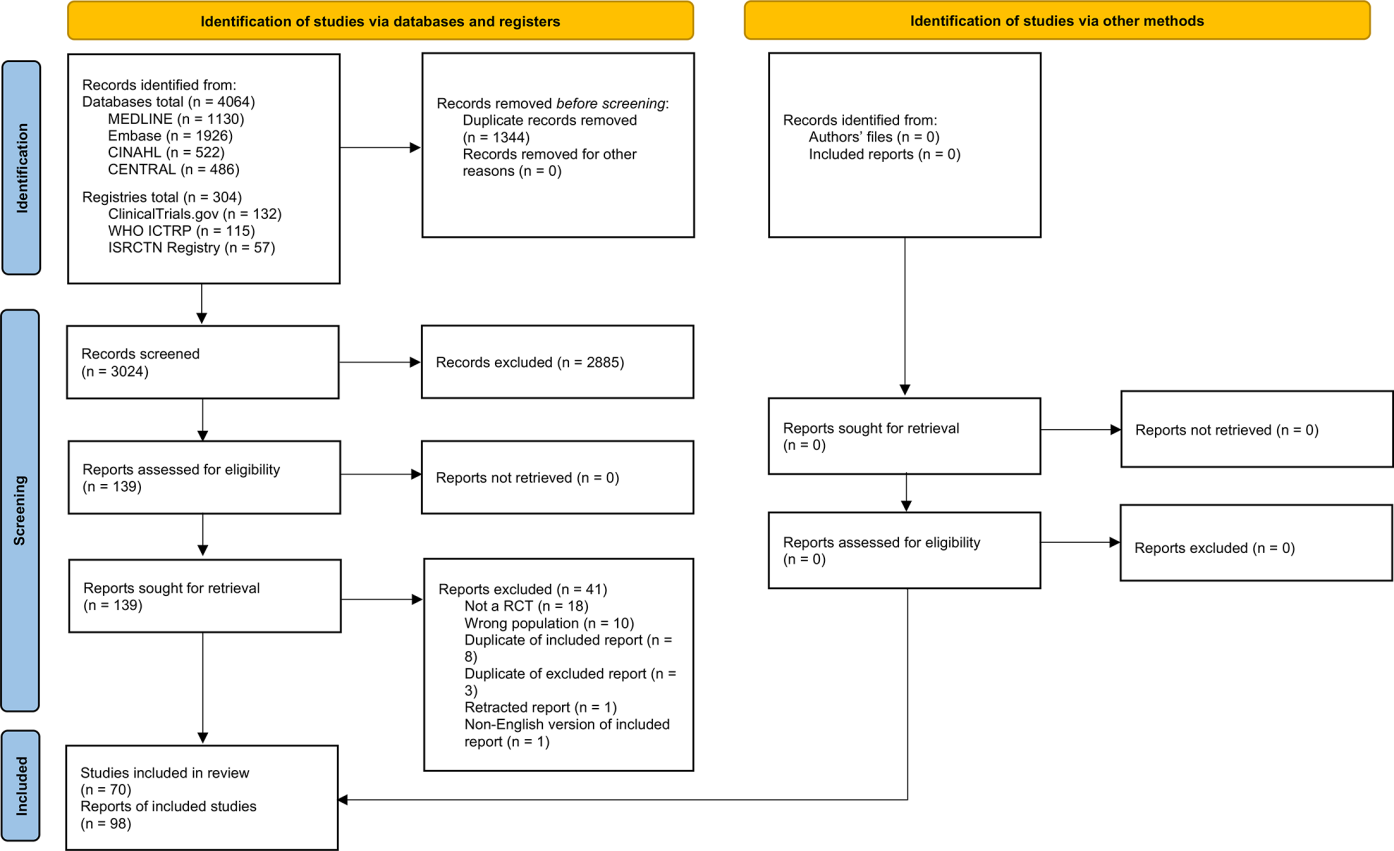
PRISMA 2020 flow diagram of the systematic review search and screening process.

The search strategy retrieved 4368 records and is summarized in Figure 1. After deduplication, 3,024 titles and abstracts remained and were screened, 139 were deemed potentially eligible and underwent full-text screening, and 98 reports of 70 studies were included in the review. Supplementary Table iii presents a list of the included studies and any linked reports, and the studies excluded at full-text screening with exclusion reasons.

### Characteristics of included studies

At the time of data extraction, 41/70 studies (59%) were complete and had disclosed results, 14/70 studies (20%) were judged complete (11 completed ≥ 12 months), but had no available results, and 15/70 studies (21%) were ongoing, two of which had published preliminary data.^29,30^ The total number of participants in completed studies was 2537 (data from 45 studies), 63% (1,281/2,087 participants, data from 36 studies) were females, and the mean sample size was 58 (SD 29; median 60 (IQR 36 to 80); data from 45 studies). In all, 36 studies (51%) evaluated surgical interventions only and 27 studies (39%) occurred in China. Additional study characteristics are presented in Table I.

**Table I.** Characteristics of included studies.

Variable	Total (n = 70), n (%)	Surgical vs surgical (n = 36), n (%)	Surgical vs non-surgical (n = 16), n (%)	Non-surgical vs non-surgical (n = 13), n (%)	Pharmacological vs pharmacological (n = 4), n (%)
**Study design**					
RCT*	61 (87)	32 (89)	13 (81)	11 (85)	4 (100)
Quasi RCT	5 (7)	3 (8)	2 (13)	-	-
Pilot/feasibility RCT	4 (6)	1 (3)	1 (6)	2 (15)	-
**Publication/registration year†**					
2020 to 2024	26 (37)	13 (36)	5 (31)	5 (38)	3 (75)
2015 to 2019*	22 (31)	13 (36)	4 (25)	4 (31)	-
2010 to 2014	14 (20)	7 (19)	3 (19)	4 (31)	-
2005 to 2009	5 (7)	1 (3)	3 (19)	-	1 (25)
2000 to 2004	1 (1)	1 (3)	-	-	-
< 2000	2 (3)	1 (3)	1 (6)	-	-
**Continent**					
Asia (n = 2 countries)					
China*	27 (39)	21 (58)	1 (6)	1 (8)	3 (75)
Israel	1 (1)	-	-	-	1 (25)
Europe (n = 11 countries)					
Finland	6 (9)	1 (3)	3 (19)	2 (15)	-
UK	6 (9)	1 (3)	2 (13)	3 (23)	-
Denmark	3 (4)	2 (6)	1 (6)	-	-
Poland	2 (3)	2 (6)	-	-	-
France	1 (1)	1 (3)	-	-	-
Germany	1 (1)	-	1 (6)	-	-
Netherlands	1 (1)	-	-	1 (8)	-
Norway	1 (1)	-	1 (6)	-	-
Sweden	1 (1)	-	1 (6)	-	-
Switzerland	1 (1)	1 (3)	-	-	-
Turkey	1 (1)	1 (3)	-	-	-
North America (n = 2 countries)					
USA	6 (9)	1 (3)	3 (19)	2 (15)	-
Canada	1 (1)	1 (3)	-	-	-
South America (n = 2 countries)					-
Brazil	4 (6)	-	2 (13)	2 (15)	-
Colombia	1 (1)	-	-	1 (8)	-
Australasia (n = 1 country)					-
Australia	3 (4)	2 (6)	-	1 (8)	-
Africa (n = 1 country)					-
Egypt	1 (1)	1 (3)	-	-	-
NR	2 (3)	1 (3)	1 (6)	-	-
**Patellar dislocation characteristics: acute or non-acute**					
Acute‡	18 (26)	2 (6)	8 (50)	8 (62)	-
Non-acute*	22 (31)	18 (50)	3 (19)	-	-
UC or NR	30 (43)	16 (44)	5 (31)	5 (38)	4 (100)
**Patellar dislocation characteristics: first time or recurrent**					
First time dislocations	21 (30)	-	12 (75)	8 (62)	1 (25)
Recurrent*	26 (37)	20 (56)	3 (19)	1 (8)	1 (25)
first time and recurrent dislocation	15 (21)	10 (28)	1 (6)	4 (31)	-
UC or NR	8 (11)	6 (17)	-	-	2 (50)

* We could not determine the interventions of one included study, so this study is not counted in the intervention columns.

† Where the main study report was a trial registry record, this was the date of registration or the date the record was first posted on the registration website.

‡ Defined as ≤ six weeks of injury or if the time since injury was not reported, but the term “acute” was used to describe the patellar dislocation.

NR, not reported; RCT, randomized controlled trial; UC, unclear.

## Primary objectives

**Outcome domains:** In total, 141 unique outcomes were identified from the included studies (mean 9.7 (SD 5.9); median per study 9 (IQR 5 to 14)). The three most common were a recurrent ipsilateral patellar dislocation (55/70 studies), the Kujala Patellofemoral Score^31^ (50/70 studies), and adverse event(s) (38/70 studies). Overall, 81 (57%) of these outcomes were measured only once among included studies. Supplementary Table iv contains the extracted verbatim outcomes, the corresponding unique outcome, and the number of studies measuring each unique outcome.

Unique outcomes (and individual PROM items where relevant) mapped onto 66 second-level domains of the WHO ICF framework (see Table II), the majority (56%, 593/1,052) in the ‘activities and participation’ domain. The unique outcomes not covered in the WHO ICF framework included, but were not limited to, adverse events, later surgery, and outcomes related to satisfaction, health-resource use, and activity where the type of activity was unspecified and could not be inferred.

**Table II.** Unique outcomes mapped onto WHO ICF framework.

Domain	Frequency measured, n (%)
**Body functions**	296 (28.1)
b126 Temperament and personality functions	7 (0.7)
b130 Energy and drive functions	6 (0.6)
b134 Sleep functions	4 (0.4)
b140 Attention functions	1 (0.1)
b152 Emotional functions	76 (7.2)
b160 Thought functions	1 (0.1)
b164 Higher-level cognitive functions	4 (0.4)
b180 Experience of self and time functions	3 (0.3)
b270 Sensory functions related to temperature and other stimuli	2 (0.2)
b280 Sensation of pain	76 (7.2)
b430 Haematological system functions	2 (0.2)
b710 Mobility of joint functions	43 (4.1)
b715 Stability of joint functions	29 (2.8)
b729 Functions of the joints and bones, other specified and unspecified	9 (0.9)
b730 Muscle power functions	19 (1.8)
b740 Muscle endurance functions	4 (0.4)
b760 Control of voluntary movement functions	2 (0.2)
b770 Gait pattern functions	7 (0.7)
b798 Neuromusculoskeletal and movement-related functions, other specified	1 (0.1)
**Body structures**	36 (3.4)
s750 Structure of lower limb	36 (3.4)
**Activities and participation**	593 (56.4)
d177 Making decisions	1 (0.1)
d220 Undertaking multiple tasks	1 (0.1)
d230 Carrying out daily routine	26 (2.5)
d299 General tasks and demands, unspecified	3 (0.3)
d399 Communication, unspecified	1 (0.1)
d410 Changing basic body position	54 (5.1)
d415 Maintaining a body position	26 (2.5)
d420 Transferring oneself	3 (0.3)
d430 Lifting and carrying objects	21 (2.0)
d435 Moving objects with lower limbs	3 (0.3)
d445 Hand and arm use	5 (0.5)
d449 Carrying, moving and handling objects, other specified and unspecified	1 (0.1)
d450 Walking	47 (4.5)
d455 Moving around	127 (12.1)
d460 Moving around in different locations	4 (0.4)
d470 Using transportation	1 (0.1)
d475 Driving	3 (0.3)
d499 Mobility, unspecified	1 (0.1)
d510 Washing oneself	3 (0.3)
d540 Dressing	7 (0.7)
d570 Looking after one’s health	3 (0.3)
d620 Acquisition of goods and services	2 (0.2)
d630 Preparing meals	2 (0.2)
d640 Doing housework	25 (2.4)
d750 Informal social relationships	1 (0.1)
d760 Family relationships	2 (0.2)
d770 Intimate relationships	2 (0.2)
d779 Particular interpersonal relationships, other specified and unspecified	1 (0.1)
d799 Interpersonal interactions and relationships, unspecified	1 (0.1)
d820 School education	7 (0.7)
d839 Education, other specified and unspecified	3 (0.3)
d850 Remunerative employment	26 (2.5)
d855 Non-remunerative employment	8 (0.8)
d859 Work and employment, other specified and unspecified	5 (0.5)
d870 Economic self-sufficiency	2 (0.2)
d910 Community life	2 (0.2)
d920 Recreation and leisure	163 (15.5)
**Environmental factors**	36 (3.4)
e120 Products and technology for personal indoor and outdoor mobility and transportation	3 (0.3)
e155 Design, construction and building products and technology of buildings for private use	1 (0.1)
e298 Natural environment and human-made changes to environment, other specified	2 (0.2)
e299 Natural environment and human-made changes to environment, unspecified	1 (0.1)
e320 Friends	1 (0.1)
e450 Individual attitudes of health professionals	4 (0.4)
e535 Communication services, systems and policies	1 (0.1)
e540 Transportation services, systems and policies	1 (0.1)
e580 Health services, systems and policies	22 (2.1)
**Other**	91 (8.7)
Not definable	6 (0.6)
Not definable – general health	10 (1.0)
Not definable – physical health	7 (0.7)
Not definable – quality of life	1 (0.1)
Personal factor	8 (0.8)
Not covered	59 (5.6)

WHO ICF, World Health Organization International Classification of Functioning, Disability and Health.

**Outcome measurement instruments:** In all, 47 different PROMs met the criteria for synthesis with 28 (60%) PROMs measured only once among included studies. The most commonly measured PROM was the Kujala Patellofemoral Score,^31^ used by 50/70 (71%) included studies. Table III shows the PROMs reported in included RCTs and the frequency they were measured.

**Table III.** PROMs in included studies.

Variable	Included studies (n = 70) that used each PROM, n (%)
Region specific PROMs* (n = 25)	
Kujala Patellofemoral Score	50 (71)
Lysholm Knee Scoring Scale	33 (47)
Tegner Activity Score	25 (36)
IKDC Subjective Knee Form	15 (21)
KOOS knee-related quality of life subscale, KOOS sport and recreation subscale	9 (13)
KOOS activities of daily living subscale, KOOS pain subscale, KOOS symptoms subscale	8 (11)
KOOS4, Lower Limb Functional Scale	2 (3)
Cincinnati Sports Activity Scale, Fulkerson scale, KOOS-Child pain subscale, KOOS-Child other symptoms subscale, KOOS-Child activities in daily living subscale, KOOS-Child function in sports and play subscale, KOOS-Child knee-related quality of life subscale, KOOS patellofemoral pain and osteoarthritis subscale, modified Cincinnati Knee Rating System, modified Functional Index questionnaire, modified Hughston Clinic Questionnaire, modified Marx activity scale, Oxford Knee Score – Activity and Participation Questionnaire, Pedi-IKDC	1 (1)
**Condition specific PROMs (n = 6)**	
Norwich Patellar Instability Score	8 (11)
Banff Patella Instability Instrument	5 (7)
Banff Patella Instability Instrument 2.0	2 (3)
Anterior Cruciate Ligament Quality of Life score, Larsen and Lauridsen criteria, Victorian Institute of Sport Assessment-Patella	1 (1)
**Generic PROMs (n = 5)**	
EQ-5D-5L	4 (6)
12-Item Short-Form Health Survey	3 (4)
WHOQOL-BREF	2 (3)
EQ-5D-Y, 36-Item Short-Form Health Survey	1 (1)
**Other PROMs (n = 11)**	
VAS for pain	15 (21)
NRS for pain	6 (9)
Cincinnati Occupational Rating Scale, Donor Site Morbidity questionnaire, Hospital for Special Surgery Paediatric Functional Activity Brief Scale, Inpatient satisfaction questionnaire, VAS for patellar insecurity, PROMIS Depression, PROMIS Pain Interference, PROMIS Physical Function, PROMIS Short Form - Satisfaction with Social Roles and Activities 4 a	1 (1)

* Individual subscales of the KOOS and KOOS-Child counted separately as not all subscales were measured in studies that used these PROMs.

IKDC, International Knee Documentation Committee; KOOS, Knee injury and Osteoarthritis Outcome Score; KOOS4, average of four of the five KOOS subscales (pain, other symptoms, function in sports and recreational activities, and knee-related quality of life); KOOS-Child, Knee injury and Osteoarthritis Outcome Score for children; NRS, Numeric Rating Scale; Pedi-IKDC, Modified International Knee Documentation Committee Subjective Knee Form in children with knee disorders; PROM, patient-reported outcome measure; PROMIS, Patient-Reported Outcomes Measurement Information System; VAS, visual analogue scale; WHOQOL-BREF, World Health Organization Quality of Life-BREF.

**Fig. 2:**
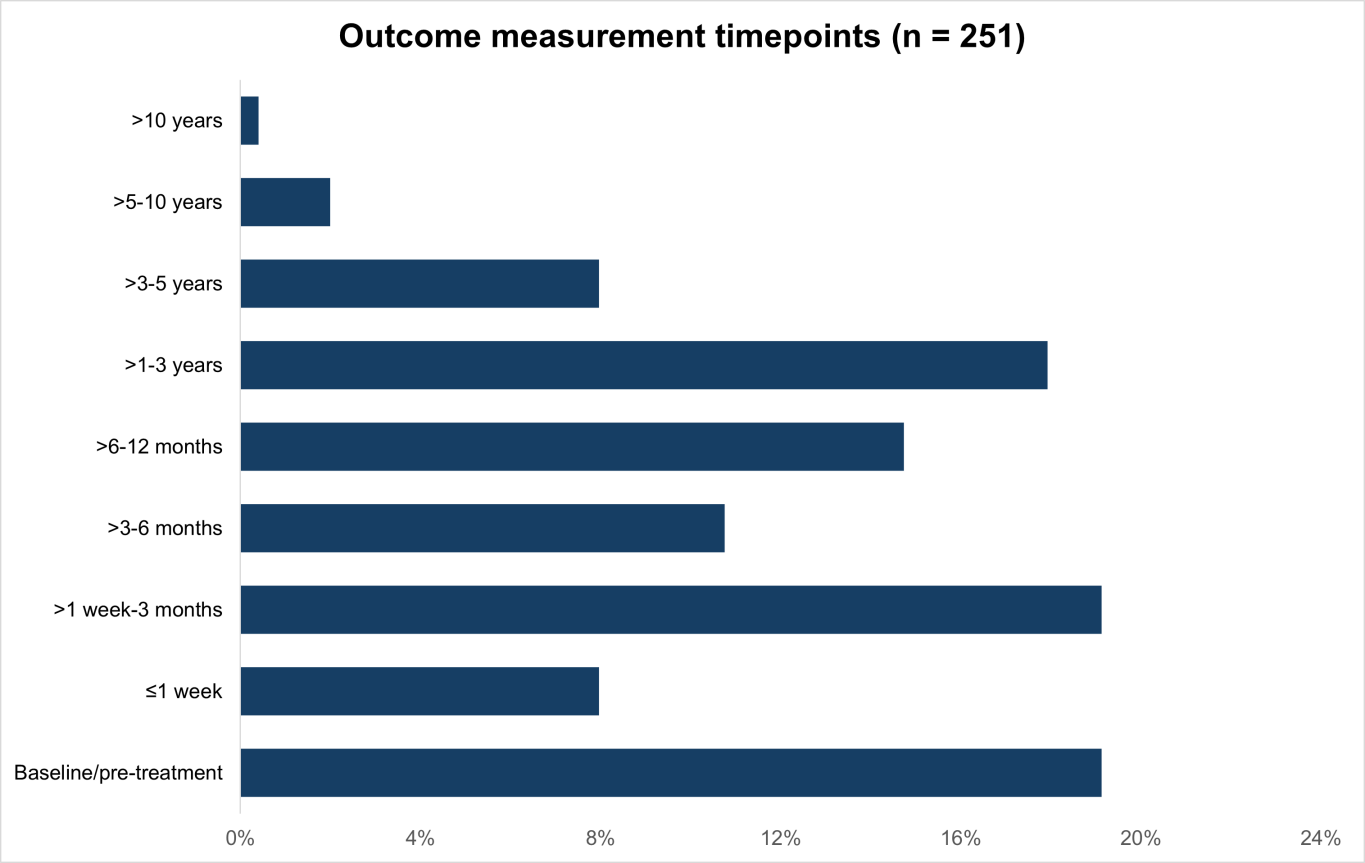
Outcome measurement timepoints in included studies.

**Outcome measurement timepoints:** There was wide variation in the outcome measurement timepoints used in included studies as shown by Figure 2. Three studies did not report the timing of outcome measurement; the main study report for these was a registration on the Chinese Clinical Trial Registry. Primary outcome measurement timepoints were also synthesised and are reported in the next section.

## Secondary objectives

**Primary outcomes:** In total, 31 different primary outcomes were identified in 47/70 included studies (67%), the most common being the Kujala Patellofemoral Score^31^ (18/47 studies). Twenty-nine studies (62%) had one primary outcome, but 18 (38%) reported multiple primary outcomes (median per study 1; IQR 1 to 2). Fourteen primary outcomes (45%) were used only once across included studies. Table IV shows the primary outcomes in included studies and the frequency they were measured.

**Table IV.** Primary outcomes in included studies.

Variable	Studies (n = 47) that used it as a primary outcome, n (%)
**PROMs (n = 9)**	
Kujala Patellofemoral Score	18 (38)
Lysholm Knee Scoring Scale	11 (23)
Tegner Activity Score	5 (11)
IKDC Subjective Knee Form	3 (6)
KOOS4, Norwich Patellar Instability Score, VAS unspecified	2 (4)
Banff Patella Instability Instrument, Banff Patella Instability Instrument 2.0	1 (2)
**Adverse events (n = 5)**	
Recurrent ipsilateral patellar dislocation	13 (28)
Composite outcome of recurrent ipsilateral patellar dislocation and subluxation	4 (9)
Ipsilateral patellar subluxation, later surgery	2 (4)
Time to recurrent ipsilateral patellar dislocation	1 (2)
**Radiological outcomes (n = 5)**	
Patellar tilt	3 (6)
Tibial tuberosity to trochlear groove distance, patellar height (Insall-Salvati ratio and Caton-Deschamps index)	2 (4)
Quadriceps angle, surgical femoral tunnel location (distance between the femoral tunnel centre and Schottle’s point)	1 (2)
**Physical performance test (n = 1)**	
Objective knee extensor muscle strength	3 (6)
**Biomarkers of cartilage degradation (n = 5)**	
MRI T1 rho relaxation times, MRI T2 relaxation times, urinary C-terminal cross-linked telopeptide of type II collagen, neoepitope of type I collagen cleavage, neoepitope of type II collagen cleavage	1 (2)
**Other (n = 1)**	
Medial retinaculum healing time	1 (2)
**Physical examination (n = 2)**	
Knee joint swelling, knee flexion and extension passive ROM	1 (2)
**Blood biochemistry (n = 2)**	
Fibrinogen concentration, fibrin concentration	1 (2)

IKDC, International Knee Documentation Committee; KOOS4, average of four of the five KOOS subscales (pain, othersymptoms, function in sports and recreational activities, and knee-related quality of life); KOOS, Knee injury and Osteoarthritis Outcome Score; MRI, magnetic resonance imaging; PROM, patient-reported outcome measure; ROM, range of movement; VAS, visual analogue scale.

**Fig. 3:**
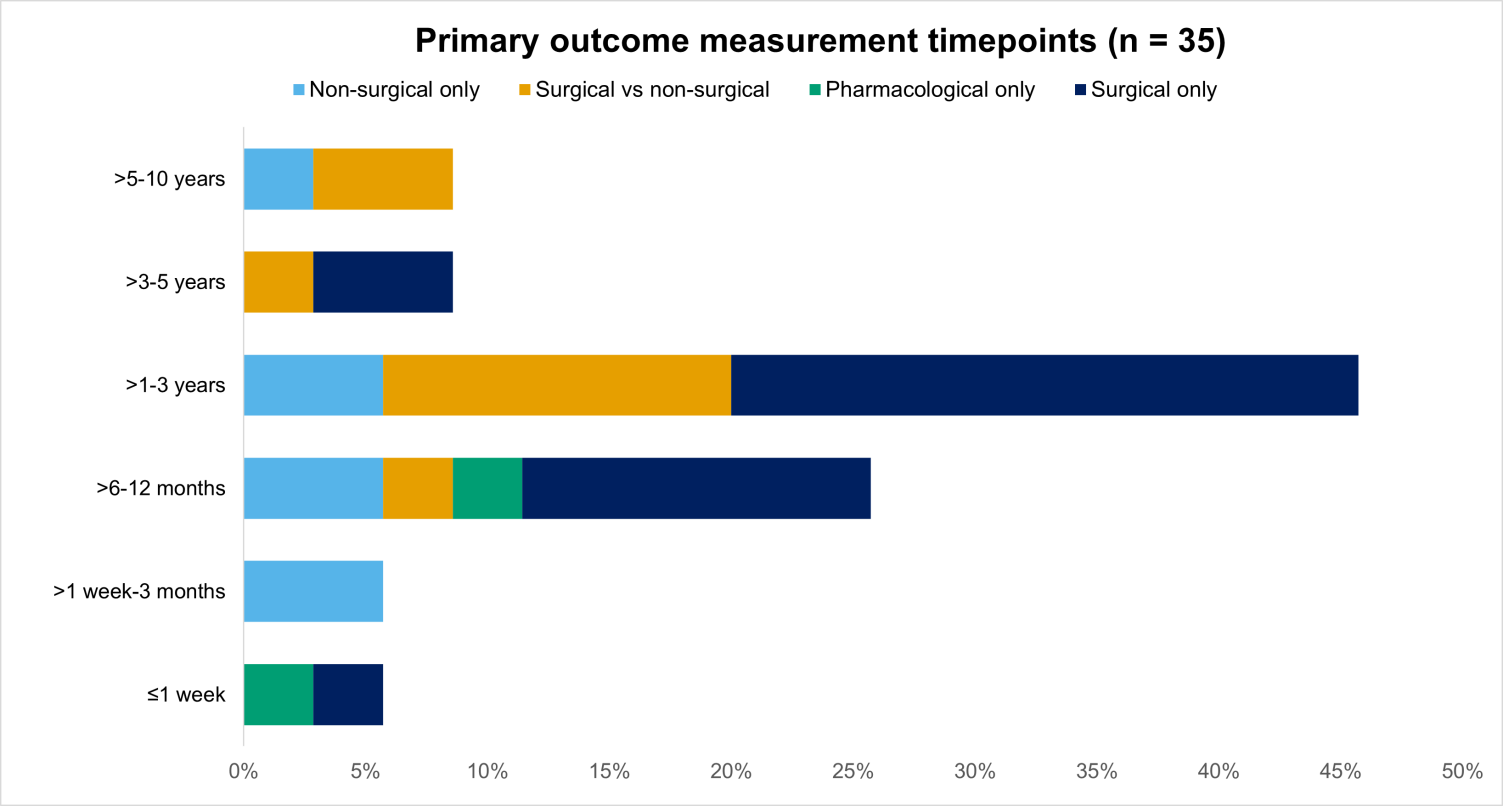
Primary outcome measurement timepoints by evaluated interventions.

A primary outcome measurement timepoint could be determined in 31/47 studies (66%). In one of these studies a different measurement timepoint was used for one of the four primary outcomes, and in three other studies the measurement timepoint was reported for some but not all primary outcomes. Because the decision about the primary outcome measurement timepoint is often influenced by when the evaluated interventions are anticipated to have greatest therapeutic benefit,^32^Figure 3 displays the primary outcome measurement timepoints by evaluated intervention.

**How a recurrent patellar dislocation was defined as an outcome:** Overall, 55 studies (79%) measured a recurrent ipsilateral patellar dislocation as an outcome (six studies also measured contralateral dislocations), but only 17 (31%) reported an outcome definition i.e., the criteria required for a recurrent ipsilateral dislocation event to be recorded (see Supplementary Table v). The most commonly used (by seven studies) definitions were those that considered a dislocation to have occurred if it was reported by a participant and/or healthcare professional.

## Discussion

This systematic review found high variability in the outcome domains, PROMs, outcome measurement timepoints, and primary outcomes used in RCTs evaluating patellar dislocation treatments. This variability in what is measured, when, and how, makes outcome selection challenging for researchers designing future RCTs. The meta-analysis of results from individual trials is also affected which is important because RCTs of this condition are frequently underpowered to detect important but rare events, such as recurrent patellar dislocations or later surgery. These findings underline the need for a COS for RCTs in this area.

Some outcome variability in included studies was expected given their different interventions, participant populations, and evaluation focus (i.e. effectiveness versus efficacy). However, over half of unique outcomes and PROMs were measured in a single study only. This appears excessive, even accounting for included studies’ different characteristics. However, these findings are not unique to this clinical condition. For example, a systematic review of fractures and joint injuries of the hand identified 639 unique outcomes with 71% of these assessed only once across included studies.^11^

The reported variability in primary outcomes underlines the need for a COS in patellar dislocation. Ideally, the primary outcome should be the outcome that is most important to relevant stakeholders.^33^ Therefore, some consistency in primary outcome selection should be observed notwithstanding justifiable reasons for variation (e.g. due the intended effects of different interventions).^32^ However, we found that the primary outcomes in included RCTs evaluated a wide variety of domains and nearly half of primary outcomes were measured just once across included studies.

The most commonly measured outcome was a recurrent ipsilateral patellar dislocation. However, over two thirds of studies that used this did not report how it was defined. This poor reporting affects the interpretation of results. When different definitions (and thresholds) are used for harms, the number of events recorded can be affected. For example, a higher number of patellar dislocations would be anticipated in a study where dislocations were defined as self-reported versus radiological evidence of dislocation. This is particularly important for this injury where dislocations are often self-reduced by patients, diagnosis can be challenging, and some patients may not seek healthcare after a new dislocation episode. When reporting future RCTs, researchers should adhere to the Consolidated Standards of Reporting Trials (CONSORT) guidelines on reporting outcomes and harms^32,34^ to help interpret individual trial results and to facilitate meta-analyses.

Though not the focus of this systematic review, the poor design and reporting of many included studies deserves comment. Registering studies and making results publicly available is required by the Declaration of Helsinki.^35^ Yet, many included studies that were recently published did not have a corresponding trial registry record, and many studies had not disclosed results, long after their completion date. When trial results are unpublished, healthcare decisions are not fully informed by the available evidence. Trials may also be unnecessarily repeated, wasting research resources.^36^ The primary outcome could also not be determined in one third of included studies, and in a third of studies with a primary outcome the measurement timepoint was not stated even though this should be reported according to CONSORT guidelines.^33^ Guidance on designing and reporting RCTs is freely available and should be consulted by those conducting RCTs in future.^37,38^

### Strengths and limitations

This study has a number of strengths. Although guidance on identifying unique outcomes exists,^26^ this is sparse. We have therefore transparently reported our methods and results so that readers can clearly understand and appraise what was done. Our search strategy was comprehensive and eligibility criteria were broad, making this a comprehensive review of outcomes used in RCTs in this area. To minimize errors, record screening, data extraction, and the identification and naming of unique outcomes was completed by two reviewers independently.

However, there were also some limitations. First, we did not contact study authors for missing outcome related data. However, contacting study authors for missing data for systematic reviews is often unsuccessful.^39^ Supporting this finding, only 2/11 people replied to emails about possible multiple study reports in this review. Second, unlike similar systematic reviews of outcomes,^10,11^ we excluded observational studies which could be seen as a limitation. However, subsequent steps in the COS development process are likely to detect any relevant outcomes that were missed.

In conclusion, this systematic review found high variability in the outcome domains, PROMs, primary outcomes, and outcome measurement timepoints used in RCTs involving people with patellar dislocations. This makes outcome selection for future RCTs challenging, and limits the comparison and meta-analysis of results from existing RCTs. The findings underline the need to develop a COS to reduce outcome heterogeneity in RCTs in this area. The development of a COS for trials of people with patellar dislocations is underway and is being informed by this systematic review’s findings.

**Take home message**

- This systematic review showed there is high outcome variability in randomized controlled trials (RCTs) evaluating treatments for people with a patellar dislocation. This makes outcome selection for future trials challenging, and also limits the comparison and meta-analysis of results from different trials.

- Developing a core outcome set is needed to address outcome variability in RCTs in this area.

## Data Availability

The data that support the findings for this study are available to other researchers from the corresponding author upon reasonable request.
